# The Swedish National Quality Register for Repetitive Transcranial Magnetic Stimulation

**DOI:** 10.1111/acps.70082

**Published:** 2026-02-26

**Authors:** S. Melker Hagsäter, Axel Nordenskjöld, Pia Nordanskog, Johan Lundberg, Carl Johan Ekman, Fredrik Hieronymus, Robert Bodén

**Affiliations:** ^1^ Department of Pharmacology, Institute of Neuroscience and Physiology The Sahlgrenska Academy at the University of Gothenburg Gothenburg Sweden; ^2^ Kungälv Hospital Västra Götaland Regional Council Kungälv Sweden; ^3^ Faculty of Medicine and Health, University Health Care Research Centre Örebro University Örebro Sweden; ^4^ Department of Biomedical and Clinical Sciences, Center for Social and Affective Neuroscience Linköping University Linköping Sweden; ^5^ Department of Psychiatry in Linköping, Department of Biomedical and Clinical Sciences Linköping University Linköping Sweden; ^6^ Centre for Psychiatry Research, Department of Clinical Neuroscience Karolinska Institute, Stockholm Health Care Services Region Stockholm Sweden; ^7^ Department of Medical Sciences, Clinical Psychiatry Uppsala University Uppsala Sweden

**Keywords:** depression, epidemiology, quality‐register, repetitive transcranial magnetic stimulation, theta‐burst stimulation

## Abstract

**Objective:**

Repetitive transcranial magnetic stimulation (rTMS) has been demonstrated to be an effective and well tolerated treatment for depression, and it is being investigated also for other indications. This study presents the Swedish National Quality Register for rTMS (Q‐rTMS). The registry comprises epidemiological data, data from standardized rating scales, details on treatment settings, and a registry‐specific patient questionnaire.

**Methods:**

A presentation of the Q‐rTMS including the types of data collected, the organizational structure of the register, as well as a brief overview of the volume of data accumulated between the registry's inception in 2018 and the end of 2024.

**Results:**

As of 2024, the register contained data from a total of 3083 unique individuals and 3842 treatment series, collected from 27 different rTMS providers. The most common indication for rTMS was depression (International Classification of Diseases, Tenth Revision diagnoses (ICD‐10): F32–F34, 74.1%) followed by bipolar affective disorder (ICD‐10: F31, 14.2%). The average age was 43.2 years, and 56.5% were women. The register included matched pre‐ and post‐treatment data exceeding 60% completeness for the Montgomery–Åsberg Depression Rating Scale—Self Assessment (MADRS‐S), the Clinical Global Impression–Severity (CGI‐S), and the EuroQol five‐dimensional questionnaire (EQ‐5D‐5L).

**Conclusion:**

The Q‐rTMS combines high coverage with longitudinal documentation of clinically relevant outcome measures and may hence contribute to population‐based real‐world effectiveness research in rTMS for depression.

## Key Points

- Significant outcomes
  ○ This paper presents the Swedish Q‐rTMS, describing the types of data collected and the organizational structure of the registry.
  ○ The Q‐rTMS is currently the largest population‐based registry of its kind and represents a valuable resource for real‐world effectiveness research in rTMS.
  ○ Data from the Q‐rTMS may be linked with other Swedish registries, allowing for enriched datasets and long‐term outcome follow‐up.
- Limitations
- Reporting to Q‐rTMS is voluntary, and response rates vary across the variables collected in the registry.

## 1 Introduction

### 1.1 Background

Repetitive transcranial magnetic stimulation (rTMS) is regarded as an effective treatment for depression [1] and has experienced rapid growth in popularity in recent years [2], following its Food and Drug Administration approval for the treatment of major depressive disorder in 2008 [3, 4]. rTMS is safe and well‐tolerated [1, 4], and offers a distinct complement to pharmacological and conventional therapeutic approaches, as well as to electroconvulsive therapy (ECT). Most treatment guidelines agree that rTMS should be considered an option for treatment‐resistant depression [5], with some suggesting that rTMS might also serve as a cost‐effective first‐line treatment [6]. A TMS device passes an electric current through a coil placed on the scalp of the patient. This generates a magnetic field in the coil which in turn induces an electric field within the cortex, thereby locally modulating neuronal activity and plasticity [7, 8]. Beyond depression, rTMS has been applied to other indications, including anxious depression, obsessive‐compulsive disorder, and migraine pain [9].

Although rTMS was introduced in clinical practice in Sweden as early as in the year 2000 [10], substantial growth and spread of the method was first seen after the Swedish National Board of Health and Welfare included rTMS in its treatment guidelines for depression in 2017 [11]. In response to the increasing use of rTMS, the idea of establishing a dedicated national register for rTMS was adopted. Consequently, in 2018, the Swedish National Quality Register for Repetitive Transcranial Magnetic Stimulation (Q‐rTMS) was founded as an appendix to the Swedish National Quality Register for ECT (Q‐ECT) [12]. The Q‐rTMS was built upon the existing framework of the ECT register and the aim was that the registry should serve both for healthcare quality assurance and research purposes. Participation in the Q‐rTMS is voluntary for both patients and providers.

While evidence from randomized clinical trials (RCTs) is the gold standard for establishing efficacy as well as short‐term safety, RCTs typically have neither sufficient power nor duration to detect uncommon and/or long‐term adverse events [13]. Also, RCTs tend to focus on highly selected populations which do not necessarily generalize well to the patient populations for whom the interventions are ultimately used [14]. Epidemiological studies using routinely collected health registry data—which has a long tradition in Scandinavian countries due to comprehensive centralized record keeping—overcome some of these problems. However, typical health registries lack information on illness severity as well as detailed information on the treatments administered, thereby making them susceptible to confounding‐by‐indication as well as less well‐suited for evaluating specific treatment modalities [15]. A dedicated quality register offers a middle‐ground in that variable coverage can be tailored to the specific interventions and conditions under study. And, since the registry data includes patients' unique Swedish personal identification number, data from the Q‐rTMS may be linked to data from other Swedish registries, hence making it possible to also track long‐term outcomes [15, 16, 17, 18].

From 2019 and onward, parts of the data collected in the Q‐rTMS have been presented (in Swedish) in the register's annual reports [19]. Two scientific papers exploring the outcome of rTMS for the treatment of depression have used data from the register [20, 21]. To date, no peer‐reviewed publication has, however, provided a comprehensive description of the register, its data content, and coverage.

### 1.2 Aims of the Study

The aim of this report is to provide the first comprehensive presentation of the Swedish National Quality Register for Repetitive Transcranial Magnetic Stimulation, including a description of the register's contents and an overview of data collected between 2018 and 2024.

## 2 Materials and Methods

### 2.1 Structure and Content of the Q‐rTMS Register

The Q‐rTMS is structured around a unique ID assigned to each treatment series, that is, a coherent set of treatment sessions distributed over several days/weeks. Individuals are identifiable through the recording of their Swedish personal identification number, from which birthdate and legal sex can be derived [17]. An individual may have multiple entries in the registry if they undergo recurrent rTMS treatment, with each entry recorded under a separate unique treatment series ID. All records can either be entered manually into the register by healthcare staff or imported from the electronic medical record for the individual patient. When manually entered, the input is either selected from drop‐down lists or entered as free text. For some measures, both options are available. Treatment dates are entered into the register via a calendar view, which allows for multiple treatments to be recorded on the same day. The registry also contains a registry‐specific patient questionnaire, which is distributed at the six‐month follow‐up.

Each registry record permits the entry of data across the following categories:

- Treatment settings
  ○ Reporting unit, treatment/record registration date
- Technical and procedural aspects of treatment
  ○ Protocol, stimulator, coil, localization method
  ○ Treatment days, treatment sessions per day
- Patient‐related information
  ○ Personal identity number, treatment indication, concurrent medication(s), previous rTMS/ECT, suicidal history, inpatient care
- Treatment outcomes
  ○ Standardized rating scales (see Section 2.2)
  ○ Side effects
  ○ Patient‐specific questionnaire (6‐month follow‐up)

A complete list of variables in the Q‐rTMS is provided in Supplementary S1.

### 2.2 Rating Scales in the Q‐rTMS

The Q‐rTMS collects data from several different rating scales, covering multiple aspects of health and well‐being. This includes both clinician‐rated scales and structured self‐reports. The clinician‐administered scales are the Montgomery–Åsberg Depression Rating Scale (MADRS) [22] for assessing depression severity, and the Clinical Global Impression–Severity (CGI‐S) and Clinical Global Impression–Improvement (CGI‐I) [23] scales for evaluating illness severity and improvement, respectively. The Q‐rTMS does not include information about the identity or professional background of the clinician who performed the rating. The patient‐reported outcome measures are the self‐assessment version of the Montgomery‐Åsberg Depression Rating Scale (MADRS‐S) [24] for assessing depression, the EuroQol Group 5D (EQ‐5D‐5L) [25] for evaluating quality of life and the memory item of the Comprehensive Psychopathological Rating Scale (CPRS‐MS) [26] to evaluate memory effects. CPRS‐MS was primarily included because the Q‐rTMS was built upon the existing framework of the Q‐ECT, thereby aligning with already established data collection routines. All rating scales can be entered before and/or after the end of a treatment series. For MADRS‐S, specifically, there is an option to enter additional ratings during a treatment series. Patient‐reported outcome measures are also collected at a six‐month follow‐up.

### 2.3 Data Collection

Data from all registered treatment series between January 2018 and December 2024 were retrieved from the Q‐rTMS register. To prevent duplication of indices in the analysis, any treatment series that, for example, begins in December and concludes in January of the following year is presented as having occurred entirely in January. Consequently, all treatment series concluding in 2025 were excluded from the analysis.

## 3 Results

### 3.1 Coverage of the Q‐rTMS

**FIGURE 1:**
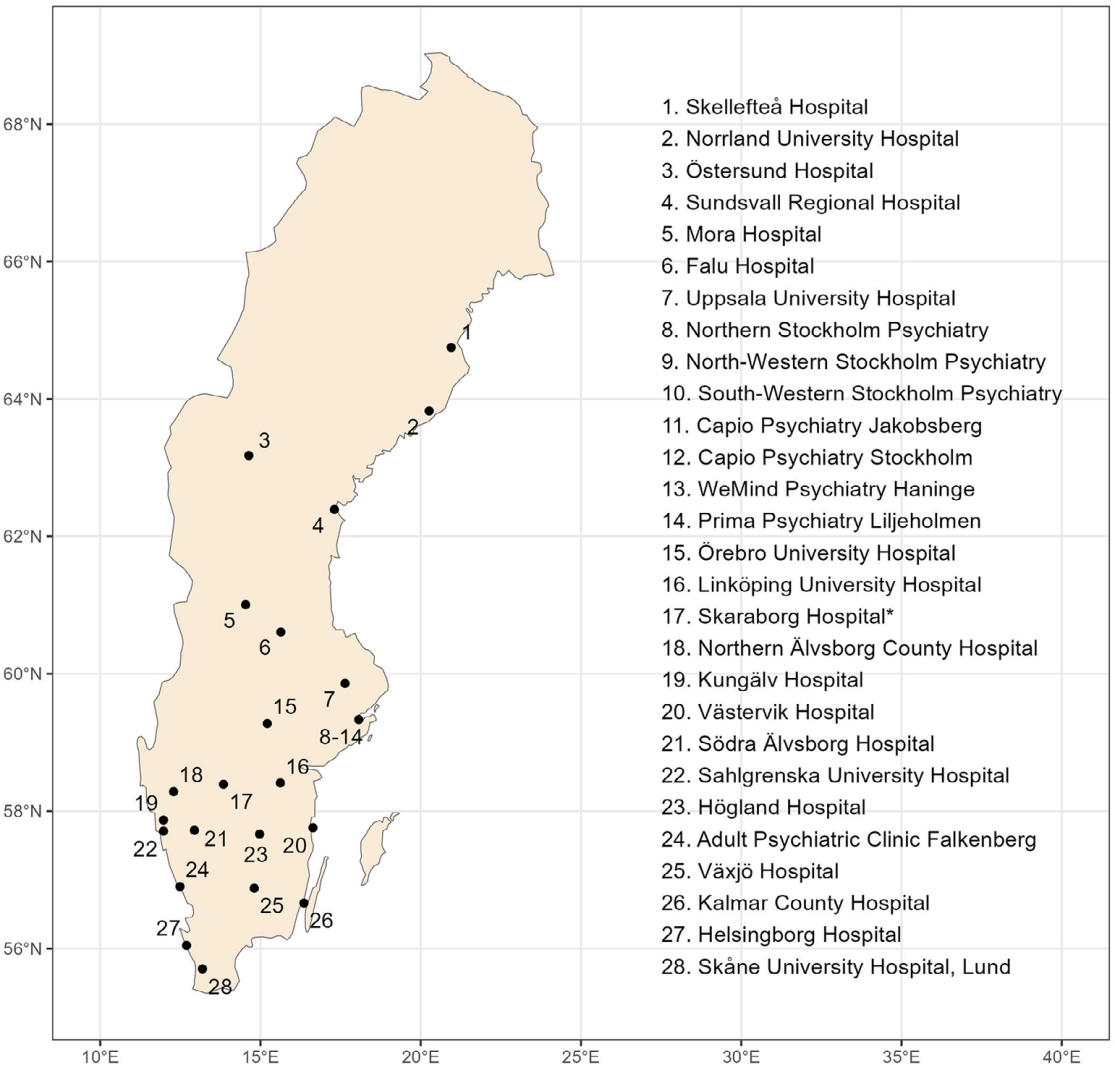
Geographical spread of sites that have contributed to the Q‐rTMS. * The rTMS unit at Skaraborg Hospital was relocated from Falkenberg to Skövde in 2022; for the purposes of this report, it is considered a single reporting entity.

All Swedish rTMS treatment providers are encouraged to contribute data to the register. All public healthcare providers and most private healthcare providers comply (Figure 1). After attachment, none of the providers have withdrawn. Between 2018 and 2024, the register collected data from a total of 3083 unique patients and 3842 treatment series (94,136 treatment sessions) (Table 1). Note that the total number of unique patients in the full 2018–2024 interval is not equivalent to the sum of unique patients for each individual year. The average age of patients remained relatively stable at around 43 years across all years. There was a slight overrepresentation of women, who accounted for 56.5% of the total sample.

**TABLE 1:** Number of rTMS treatment providers, patients, and treatment series per year.

	2018	2019	2020	2021	2022	2023	2024	2018–2024
No. of reporting rTMS providers	5	11	18	20	25	25	28	28
Of which public	5	11	16	17	21	21	24	24
Of which private	0	0	2	3	4	4	4	4
No. unique patients	56	239	460	485	595	831	799	3083
Of which women (%)	62.5	63.6	58.9	59.0	56.0	54.4	54.1	56.5
Average age (years)	43.6	43.8	43.1	42.7	43.7	43.6	43.7	43.2
(SD)	(16.9)	(16.4)	(15.1)	(14.3)	(15.1)	(15.2)	(14.9)	(15.1)
No. treatment series	57	297	505	539	650	923	871	3842

*Note*: Average age and distribution by gender are calculated per unique individual in each respective interval.

### 3.2 Indications for rTMS

Registered indications (International Classification of Diseases, Tenth Revision; ICD‐10), per treatment series in total and per year, are displayed in Table 2. Unipolar depressive episode, single episode or recurrent (F32 and F33 diagnoses), were the dominating indications for rTMS. Severe depressive episode with psychotic symptoms diagnoses (F31.5, F31.8D, F32.3 and F33.3) were rare in the sample (*n* = 21; 0.5%).

**TABLE 2:** Indications for rTMS based on diagnosis per treatment series.

Diagnosis group (ICD‐10)	%
Bipolar disorder (F31)	14.3
Mild or moderate	9.6
Severe	3.8
Unspecified and other	0.9
Depressive episode (F32)	27.2
Mild	1.3
Moderate	15.5
Severe	8.3
Unspecified & other	2.1
Recurrent depressive disorder (F33)	43.6
Mild	1.5
Moderate	26.9
Severe	12.7
Unspecified and other	2.5
Persistent mood disorders (F34)	3.4
Other diagnoses	5.7
Missing data	5.8

*Note*: Numbers are displayed as percentage of diagnoses of all treatment series (*n* = 3842). Bipolar disorder category Unspecified and other comprised ICD‐10 diagnoses F31.5 Other (*n* = 17; 0.4%), F31.9 Unspecified (*n* = 16; 0.4%), F31.6 Mixed (*n* = 1), and F31.7 In remission (*n* = 1). Depressive episode category Unspecified and other comprises ICD‐10 diagnoses F32.9 Unspecified (*n* = 78; 2.0%) and F32.8 Other (*n* = 3; 0.1%). Recurrent depressive disordered category Unspecified and other comprises ICD‐10 diagnoses F33.9 Unspecified (*n* = 85; 2.2%), F33.4 In remission (*n* = 6; 0.2%), and F33.8 Other (*n* = 4; 0.1%). The most used ICD‐10 diagnoses among Other diagnoses were F41.2 Mixed anxiety and depressive disorder (*n* = 76; 2.0%), F43.8A Burnout syndrome (*n* = 32; 0.8%), F41.1 Generalized anxiety disorder (*n* = 21; 0.5%), F25 Schizoaffective disorder (*n* = 15; 0.4%), and F41.9 Anxiety disorder, unspecified (*n* = 14; 0.4%).

### 3.3 Concurrent Medications

Registered concurrent medications by drug or drug class are displayed in Table 3. Antidepressants were the most common concurrent medication followed by antipsychotics.

**TABLE 3:** Concurrent medications during rTMS treatment.

Class of drug	Yes	No	Missing data
Antidepressants	75.8	18.9	5.3
Lithium	13.8	80.6	5.6
Lamotrigine	14.1	80.2	5.7
Valproate	1.1	93.2	5.7
Other antiepileptics	9.7	84.5	5.8
Antipsychotics	30.9	63.3	5.7
Benzodiazepines	19.0	75.1	5.9

*Note*: Numbers are displayed as percentage of all treatment series (*n* = 3842).

### 3.4 Type of Protocols, Stimulators and Coils

The Q‐rTMS collects detailed data on treatment protocol settings. For a full set of variables (in this category) see Supplementary S1. One survey question entered by healthcare staff is regarding whether Intermittent theta burst stimulation was used or not, which it was in the vast majority of treatment series (Table 4). The dominating stimulator model used was the Magventure R30, and the most used coil shape was a figure‐8 coil. Information about current direction within the coil is not collected in the register.

**TABLE 4:** Protocol, stimulator, and coil.

Name	%
Protocol	
iTBS	83.5
Not iTBS (aggregated)	16.2
Missing data	0.3
Stimulator	
Magventure R30	90.4
Magventure ×100	2.9
Magstim	0.6
Mag and More	0.6
Other stimulator	4.5
Missing data	1.0
Coil	
Figure‐8	80.8
Figure‐8 (angled)	17.4
H‐coil	0.8
Other coil	0.9
Missing data	0.1

*Note*: Numbers are displayed as percentage of all treatment series (*n* = 3842). The aggregated Not iTBS answers are “cTBS,” “other,” and “no.” The most common free text records in the “Other stimulator” category were variants of “Nexstim” (4.1%). All free text records in the “Other coil” category were variants of “Cool‐B65” (i.e., MagVenture Cool‐B65).

### 3.5 Rating Scales

The coverage of ratings scale reports before and after treatment series and at the 6‐month follow‐up are presented in Table 5. For a full list of data entries at the six‐month follow up see Supplementary S1.

**TABLE 5:** Prevalence of registered data from rating scales.

Rating scale	Before %	After %	Before and After %	Six‐month after %
Depression				
MADRS	13.6	7.7	5.5	
MADRS‐S	75.2	74.5	63.4	22.0
Illness severity				
CGI‐S	75.6	91.4	70.7	
CGI‐I		89.9		
Quality of life				
EQ‐5D‐5L	73.7	77.2	62.7	21.7
Memory				
CPRS‐MS	78.4	77.3	67.9	21.7

*Note*: Numbers are displayed as percentage of all treatment series (*n* = 3842). *Before* and *After* comprise prevalence of paired records. The included rating scales are the Montgomery–Åsberg Depression Rating Scale (MADRS), the self‐assessment version of the Montgomery–Åsberg Depression Rating Scale (MADRS‐S), the Clinical Global Impression–Severity scale (CGI‐S), the Clinical Global Impression–Improvement scale (CGI‐I), the EuroQol Group 5D scale (EQ‐5D‐5L) and the Comprehensive Psychopathological Rating Scale (CPRS‐MS). In the registry, MADRS, MADRS‐S, and EQ‐5D‐5L are adjoined by assessment date variables. For these rating scales only assessments performed within 2 weeks before first treatment are counted as *Before*, and only assessments 2 days before final treatment to 2 weeks after final treatment are counted as *After*.

## 4 Discussion

The purpose of this report is to present an overview of the Q‐rTMS registry, including the scope, volume, and type of data collected. Q‐rTMS is one of the largest rTMS registries in the world and the largest population‐based registry of its kind, making it a valuable asset for rTMS research. Real‐world registers on rTMS treatment are rare to date, and we are only aware of a few other initiatives, and none of them being population based with good coverage as Q‐rTMS. The TMS Database Registry Consortium Research Project in Japan (TReC‐J) for Future Personalized Psychiatry [27, 28], the Transcranial Magnetic Stimulation and Brain Stimulation Research Registry (TMS‐REG) [29], and the NeuroStar Advanced Therapy System Clinical Outcomes Registry, with the latter being the largest in numbers but a TMS‐provider based registry with probably a selected patient sample [30, 31]. Building on the framework of the Q‐ECT and the valuable outcomes from that register [12, 32, 33, 34, 35, 36], it is likely that much could be learned from the Q‐rTMS as well.

The Q‐rTMS register is currently growing with around 900 records annually and surpassed 4000 treatment series in total during 2025. With the number of rTMS providers in Sweden steadily increasing since 2018, the annual number of new records has increased and is expected to continue to rise. Swedish public health care is organized in 21 regional councils, rTMS, as of 2024, being available in 13 of these [19]. Based on political goals for health equity, it is likely that we will see a continued national spread of the treatment method within public health care and that these new rTMS providers will also report to Q‐rTMS thus further increasing the size of the register. Furthermore, if future medical treatment guidelines would include rTMS as a first‐line treatment [6], or at least earlier in the course of the depressive illness, a national initiative to expand the availability of rTMS within primary health care is a plausible scenario that could substantially increase the size of the Q‐rTMS dataset.

As shown in Table 1 and Figure 1, 27 rTMS providers (96%) are attached to Q‐rTMS [19], which suggests that data coverage should be high. However, to determine actual patient‐level coverage, a reference source containing data for all rTMS treatment series administered in Sweden is needed. Typically, the Swedish National Board of Health and Welfare's patient register [37]—which is mandatory for all secondary and tertiary care healthcare providers in Sweden to report to—could be used for that purpose. However, reporting to the patient registry is not well developed for rTMS and while some treatment series recorded in the patient registry are not available in the Q‐rTMS, the opposite is also true. There is thus no authoritative reference from which the true coverage can be estimated. When all treatment series recorded in either registry during 2024 were combined, the estimated patient‐level coverage for Q‐rTMS was 88% compared to 52% for the patient registry [19]. This metric has remained fairly stable (range 86%–95%) since 2019.

Depressive states accounted for roughly 90% of all treatment indications, with the most frequently registered diagnoses being Depressive disorder (F32—F34; 74.1%) and Bipolar disorder (F31; 14.2%). With respect to infrequently registered treatment indications, while some rTMS treatment series may have represented exploratory use, it is also possible that the patients' primary or persistent diagnoses were erroneously recorded in some instances.

The most common concurrent medication during rTMS was antidepressants (75.8%), which is unsurprising given that antidepressants constitute a first‐line treatment for depressive disorders. A substantial proportion of patients (30.9%) also reported concurrent use of antipsychotic medications. Since the Q‐rTMS lacks information on drug dosage and clinical indication, it is not possible—using Q‐rTMS data alone—to determine the purposes for which antipsychotic medications were prescribed (i.e., for sedative, hypnotic, adjunctive, antipsychotic, or other uses). Information on concurrent treatment changes is not collected in the registry but could be obtained from other sources, such as the Prescribed Drug Register [18].

While nearly all records contained information on protocol, stimulator, and coil (> 99%), and information on indications and concurrent medications was available in approximately 95% of cases, rating scales were less consistently recorded, with matched before‐and‐after treatment data ranging from 62.7% to 70.7% across MADRS‐S, CGI‐S, EQ‐5D, and CPRS‐MS. Clinician‐rated MADRS was an outlier with only 5.5% matched before‐and‐after treatment data, suggesting that this measure was infrequently administered; the self‐rated version (MADRS‐S) is likely favored, and the two instruments are rarely administered in parallel. Varying coverage rates is an issue since it may impact the generalizability of results obtained from the Q‐rTMS. While a detailed examination into possible explanations for the varying coverage rates lies outside the scope of this report, it is something that we intend to address in planned publications based on rating scale data from the Q‐rTMS.

Sweden has a well‐established tradition of maintaining both mandatory and voluntary health registers. Reporting to Q‐rTMS is voluntary, and no financial remuneration is provided. The absence of financial remuneration may be of particular concern for private rTMS providers, for whom the additional administrative burden could represent a significant disincentive to reporting. Continued streamlining of the Q‐rTMS reporting process is warranted to ensure that all rTMS providers, not least those in private practice, will continue to provide data.

## 5 Conclusions

This is a first presentation of the Swedish Q‐rTMS register. Up until the end of 2024, 3842 treatment series, about 9 out of 10 for depression, had been recorded in the registry. For those series, matched before‐and‐after patient‐reported outcome measures were available in 60%–70% of cases. The available data thus already vastly exceeds the sample size of a typical controlled trial and this size advantage will continue to increase as more treatment series are accrued and rTMS coverage is increased. This, in combination with the possibility for enrichment of the data when combined with data from other population‐based Swedish registries (e.g., with respect to long‐term outcomes), suggests that the Q‐rTMS can be a valuable source for researcher's looking to assess the real‐world efficacy of rTMS.

## Author Contributions

Conceptualization: all authors. Data curation: Axel Nordenskjöld. Formal analysis: Robert Bodén, Melker Hagsäter, and Fredrik Hieronymus. Investigation: Robert Bodén, Melker Hagsäter, and Fredrik Hieronymus. Project administration: Melker Hagsäter. Supervision: Robert Bodén. Visualization: Melker Hagsäter and Fredrik Hieronymus. Writing original draft: Robert Bodén, Melker Hagsäter, and Fredrik Hieronymus. Review and editing: all authors.

## Funding

The authors have nothing to report.

## Ethics Statement

The register‐based study complied with the Declaration of Helsinki and was approved by the Swedish Ethical Review Authority (2025‐05598‐02), with informed consent waived.

## Conflicts of Interest

The authors declare no conflicts of interest.

## Supporting information


**Supplementary S1.** The list of variables in the Q‐rTMS as of 2025‐11‐01. The variable names in Q‐rTMS originate from Swedish; for clarity, brief English descriptions of each variable are provided. For inquiries regarding the most up‐to‐date version, or for more detailed information, please contact the responsible researcher or the registry holder. * Treatment dates are entered into the register via a calendar view, and the variable can store multiple dates as well as multiple treatments on the same day.

## Data Availability

The data that support the findings of this study are available from the corresponding author upon reasonable request.
