# Supplementary material for: The Swedish National Quality Register for Repetitive Transcranial Magnetic Stimulation
Source: Acta Psychiatr Scand. 2026 Feb 26;153(6):649–55. doi: 10.1111/acps.70082 (PMC13124318; doi:10.1111/acps.70082)
Supplement: Supplementary file 1 — Supplementary S1. The list of variables in the Q‐rTMS as of 2025‐11‐01. The variable names in Q‐rTMS originate from Swedish; for clarity, brief English descriptions of each variable are provided. For inquiries regarding the most up‐to‐date version, or for more detailed information, please contact the responsible researcher or the registry holder. * Treatment dates are entered into the register via a calendar view, and the variable can store multiple dates as well as multiple treatments on the same day. [file ACPS-153-649-s001.docx]

*Supplementary S1.* The list of variables in the Q-rTMS as of 2025-11-01. The variable names in Q-rTMS originate from Swedish; for clarity, brief English descriptions of each variable are provided. For inquiries regarding the most up-to-date version, or for more detailed information, please contact the responsible researcher or the registry holder. * Treatment dates are entered into the register via a calendar view, and the variable can store multiple dates as well as multiple treatments on the same day.

**Q-rTMS – List of variables**

| **Description** | **Entry type** | **Variable name (Swedish)** |
| --- | --- | --- |
| Swedish personal identity number | [YY-MM-DD-NNNN] | PERSNR |
| Unique Q-rTMS patient ID | [number ID] | PAT_ID |
| Registration date | [date] | datifylld |
| Hospital | [multiple choice] | sjukhus_Värde |
| Hospital (continued) | [multiple choice] | sjukhus_Värde |
| Hospital (continued) | [multiple choice] | sjukhus_Värde |
| Reporting unit | [number ID] | enhet |
| Indication for rTMS according to ICD-10 | [multiple choice] | indikation_Värde |
| Indication for rTMS according to ICD-10 (other) | [ICD-10 code] | indikationAnnan_kod |
| Indication for rTMS (other) | [free text] | indikationFritext |
| CGI-S score before rTMS | [multiple choice] | foreCGI_Värde |
| Previous suicide attempt(s) | [multiple choice] | suicidforsok_Värde |
| Number of suicide attempts last 12 months | [multiple choice] | suicidforsok12man_Värde |
| MADRS before rTMS | [y/n/m] | foreMADRS_Värde |
| MADRS before rTMS date | [date] | foreMADRSDatum |
| MADRS score before rTMS question 1 | [number] | foreMADRSNedstamdhet_Värde |
| MADRS score before rTMS question 2 | [number] | foreMADRSSAnktGrundstamning_Värde |
| MADRS score before rTMS question 3 | [number] | foreMADRSAngestkanslor_Värde |
| MADRS score before rTMS question 4 | [number] | foreMADRSMinskadNattsomn_Värde |
| MADRS score before rTMS question 5 | [number] | foreMADRSMinskadAptit_Värde |
| MADRS score before rTMS question 6 | [number] | foreMADRSKoncsvarigheter_Värde |
| MADRS score before rTMS question 7 | [number] | foreMADRSInitiativloshet_Värde |
| MADRS score before rTMS question 8 | [number] | foreMADRSKanslomassigtEng_Värde |
| MADRS score before rTMS question 9 | [number] | foreMADRSDepTankeinnehall_Värde |
| MADRS score before rTMS question 10 | [number] | foreMADRSSjalvmordstankar_Värde |
| MADRS score before rTMS total | [number] | foreMADRSTotalpoang |
| MADRS-S before rTMS | [y/n/m] | foreMADRSS_Värde |
| MADRS-S before rTMS date | [date] | foreMADRSSDatum |
| MADRS-S score before rTMS question 1 | [number] | foreMADRSSinnesstamning_Värde |
| MADRS-S score before rTMS question 2 | [number] | foreMADRSSOroskanslor_Värde |
| MADRS-S score before rTMS question 3 | [number] | foreMADRSSSomn_Värde |
| MADRS-S score before rTMS question 4 | [number] | foreMADRSSMatlust_Värde |
| MADRS-S score before rTMS question 5 | [number] | foreMADRSSKoncformaga_Värde |
| MADRS-S score before rTMS question 6 | [number] | foreMADRSSInitiativformaga_Värde |
| MADRS-S score before rTMS question 7 | [number] | foreMADRSSKanslomassigtEng_Värde |
| MADRS-S score before rTMS question 8 | [number] | foreMADRSSPessimism_Värde |
| MADRS-S score before rTMS question 9 | [number] | foreMADRSSLivslust_Värde |
| MADRS-S score before rTMS total | [number] | foreMADRSSTotalpoang |
| CPRS-MS score before rTMS | [number] | foreMinnesstorning_Värde |
| EQ-5D-5L before rTMS | [y/n/m] | foreEQ5D_Värde |
| EQ-5D-5L before rTMS | [date] | foreEQ5DDatum |
| EQ-5D score before rTMS question 1 | [number] | foreEQ5DRorlighet_Värde |
| EQ-5D score before rTMS question 2 | [number] | foreEQ5DHygien_Värde |
| EQ-5D score before rTMS question 3 | [number] | foreEQ5DHuvudsakAktiviteter_Värde |
| EQ-5D score before rTMS question 4 | [number] | foreEQ5DSmartorBesvar_Värde |
| EQ-5D score before rTMS question 5 | [number] | foreEQ5DOroNedstamdhet_Värde |
| EQ-VAS score before rTMS | [number] | foreEQ5DHalsotillstand |
| Type of care at first rTMS | [multiple choice] | vardform_Värde |
| Voluntary care | [y/n/m] | vardasFrivilligt_Värde |
| Previous rTMS | [y/n/m] | tidigareRTMS_Värde |
| Previous ECT | [y/n/m] | tidigareECT_Värde |
| Date of rTMS treatment * | [date] | behTillfalle |
| Date of first rTMS treatment session | [date] | forstaBehTillfalle |
| Date of last rTMS treatment session | [date] | sistaBehTillfalle |
| Number of rTMS treatment sessions | [number] | antalBehTillfalle |
| Stimulator | [multiple choice] | stimulator_Värde |
| Stimulator (other) | [free text] | stimulatorAnnan |
| Coil | [multiple choice] | spoltyp_Värde |
| Coil (other) | [free text] | spoltypAnnat |
| Theta burst | [multiple choice] | thetaburstProtokoll_Värde |
| Theta burst (other) | [free text] | thetaburstannan |
| Localization | [multiple choice] | lokalisation_Värde |
| Localization (other) | [free text] | lokalisationAnnat |
| Localization method | [multiple choice] | lokalisationMetod_Värde |
| Localization method (other) | [free text] | lokalisationMetodAnnat |
| Motor threshold identification method | [multiple choice] | motortroskelMetod_Värde |
| Motor threshold identification method (other) | [free text] | motortroskelMetodAnnat |
| Motor threshold (%) | [number] | motortroskel |
| Titration | [y/n/m] | titrering_Värde |
| Treatment intensity reached (which session) | [number] | behandlingsnummer |
| Treatment intensity (% of motor threshold) | [number] | sistaStyrka |
| Frequency (Hz) | [number] | dosFrekvens |
| Number of rTMS pulses per pulse train | [number] | dosPuls |
| Time between rTMS pulse trains (s) | [number] | dosTidPulstag |
| Number of rTMS pulse trains | [number] | dosAntalPulstag |
| Total number of rTMS pulses | [number] | dosTotAntalPulser |
| Total treatment duration (min) | [number] | dosTotBehDuration |
| Total treatment duration (s) | [number] | dosTotBehDurationSekunder |
| Antidepressant treatment | [y/n/m] | antiDep_Värde |
| Lithium treatment | [y/n/m] | litium_Värde |
| Lamotrigine treatment | [y/n/m] | lamotrigin_Värde |
| Valproate treatment | [y/n/m] | valproat_Värde |
| Benzodiazepine treatment | [y/n/m] | bensodiazepin_Värde |
| Other antiepileptic drug treatment | [y/n/m] | antiEp_Värde |
| Antipsychotic treatment | [y/n/m] | antiPsyk_Värde |
| rTMS treatment planned | [y/n/m] | planFortsattRTMS_Värde |
| ECT treatment planned | [y/n/m] | planECT_Värde |
| Systemic therapeutic treatment planned | [y/n/m] | planSysPsykBeh_Värde |
| Side effect(s) | [y/n/m] | biverkanKomplikation_Värde |
| Side effect | [multiple choice] | biverkanKomplikationLista_Värde |
| Side effect (other) | [free text] | biverkanKomplikationAnnan |
| Reason for discontinued treatment | [multiple choice] | orsakAvslut_Värde |
| Reason for discontinued treatment | [free text] | orsakAvslutAnnan |
| CGI-S score during rTMS | [multiple choice] | underCGI_Värde |
| CGI-S during rTMS date | [date] | underCGISkattning |
| MADRS-S during rTMS | [y/n/m] | underMADRSS_Värde |
| MADRS-S during rTMS date | [date] | underMADRSSDatum |
| MADRS-S score during rTMS question 1 | [number] | underMADRSSinnesstamning_Värde |
| MADRS-S score during rTMS question 2 | [number] | underMADRSSOroskanslor_Värde |
| MADRS-S score during rTMS question 3 | [number] | underMADRSSSomn_Värde |
| MADRS-S score during rTMS question 4 | [number] | underMADRSSMatlust_Värde |
| MADRS-S score during rTMS question 5 | [number] | underMADRSSKoncformaga_Värde |
| MADRS-S score during rTMS question 6 | [number] | underMADRSSInitiativformaga_Värde |
| MADRS-S score during rTMS question 7 | [number] | underMADRSSKanslomassigtEng_Värde |
| MADRS-S score during rTMS question 8 | [number] | underMADRSSPessimism_Värde |
| MADRS-S score during rTMS question 9 | [number] | underMADRSSLivslust_Värde |
| MADRS-S score during rTMS total | [number] | underMADRSSTotalpoang |
| CGI-S score after rTMS | [multiple choice] | efterCGI_Värde |
| CGI-I score after rTMS | [multiple choice] | kliniskBedomning_Värde |
| MADRS after rTMS | [y/n/m] | efterMADRS_Värde |
| MADRS after rTMS date | [date] | efterMADRSDatum |
| MADRS score after rTMS question 1 | [number] | efterMADRSNedstamdhet_Värde |
| MADRS score after rTMS question 2 | [number] | efterMADRSSAnktGrundstamning_Värde |
| MADRS score after rTMS question 3 | [number] | efterMADRSAngestkanslor_Värde |
| MADRS score after rTMS question 4 | [number] | efterMADRSMinskadNattsomn_Värde |
| MADRS score after rTMS question 5 | [number] | efterMADRSMinskadAptit_Värde |
| MADRS score after rTMS question 6 | [number] | efterMADRSKoncsvarigheter_Värde |
| MADRS score after rTMS question 7 | [number] | efterMADRSInitiativloshet_Värde |
| MADRS score after rTMS question 8 | [number] | efterMADRSKanslomassigtEng_Värde |
| MADRS score after rTMS question 9 | [number] | efterMADRSDepTankeinnehall_Värde |
| MADRS score after rTMS question 10 | [number] | efterMADRSSjalvmordstankar_Värde |
| MADRS score after rTMS total | [number] | efterMADRSTotalpoang |
| MADRS-S after rTMS | [y/n/m] | efterMADRSS_Värde |
| MADRS-S after rTMS date | [date] | efterMADRSSDatum |
| MADRS-S score after rTMS question 1 | [number] | efterMADRSSinnesstamning_Värde |
| MADRS-S score after rTMS question 2 | [number] | efterMADRSSOroskanslor_Värde |
| MADRS-S score after rTMS question 3 | [number] | efterMADRSSSomn_Värde |
| MADRS-S score after rTMS question 4 | [number] | efterMADRSSMatlust_Värde |
| MADRS-S score after rTMS question 5 | [number] | efterMADRSSKoncformaga_Värde |
| MADRS-S score after rTMS question 6 | [number] | efterMADRSSInitiativformaga_Värde |
| MADRS-S score after rTMS question 7 | [number] | efterMADRSSKanslomassigtEng_Värde |
| MADRS-S score after rTMS question 8 | [number] | efterMADRSSPessimism_Värde |
| MADRS-S score after rTMS question 9 | [number] | efterMADRSSLivslust_Värde |
| MADRS-S score after rTMS total | [number] | efterMADRSSTotalpoang |
| CPRS-MS score after rTMS | [number] | efterMinnesstorning_Värde |
| EQ-5D-5L after rTMS | [y/n/m] | efterEQ5D_Värde |
| EQ-5D-5L after rTMS | [date] | efterEQ5DDatum |
| EQ-5D score after rTMS question 1 | [number] | efterEQ5DRorlighet_Värde |
| EQ-5D score after rTMS question 2 | [number] | efterEQ5DHygien_Värde |
| EQ-5D score after rTMS question 3 | [number] | efterEQ5DHuvudsakAktiviteter_Värde |
| EQ-5D score after rTMS question 4 | [number] | efterEQ5DSmartorBesvar_Värde |
| EQ-5D score after rTMS question 5 | [number] | efterEQ5DOroNedstamdhet_Värde |
| EQ-VAS score after rTMS | [number] | efterEQ5DHalsotillstand |
| Patient can consider rTMS treatment again | [multiple choice] | rtmsIgen_Värde |
| Other information or commentary to the register | [y/n] | annanInfo_Värde |
| Other information or commentary to the register | [free text] | annanInfoFritext |
| **Variables collected at six-month follow-up below** | |  |
| Six-month follow-up registration date | [date] | uppfDatifylld |
| Six-month follow-up patient questionnaire performed | [y/n] | patientenkatBesvarad_Värde |
| Six-month follow-up patient questionnaire date performed | [date] | patSvarDatRTMS |
| Q: Do you feel that you have received sufficient information about rTMS? | [multiple choice] | tillrackligInfo_Värde |
| Q: How have you been informed about rTMS? | [multiple choice] | infoMetod_Värde |
| Q: Do you believe that the rTMS treatment helped you? | [multiple choice] | hjalpteRTMS_Värde |
| Q: Did you experience any side effect(s) during the time you received rTMS treatment? | [multiple choice] | besvarRTMS_Värde |
| Q: If yes, describe the problem(s) | [free text] | besvarFritext |
| Q: How long did the problem(s) persist? | [multiple choice] | besvarLangd_Värde |
| Q: Would you consider rTMS treatment again under similar medical circumstances? | [multiple choice] | rtmsIgenUppf_Värde |
| Q: Have you received antidepressant treatment during the last 6 months? | [y/n] | q09_antidep |
| Q: Have you received Lithium treatment during the last 6 months? | [y/n] | q09_lithium |
| Q: Have you received CBT treatment during the last 6 months? | [y/n] | q09_kbt |
| Q: Have you received any other treatment during the last 6 months? | [y/n] | q09_annan |
| Q: Have you received any kind of treatment during the last 6 months? | [y/n] | q09_ingen |
| Q: Would you like to be contacted by the rTMS unit where you received your treatment? | [y/n] | fragorKontaktEnhet_Värde |
| Other comment(s) | [free text] | ovrigKommentar |
| MADRS-S score 6 mos after rTMS question 1 | [number] | uppfMADRSSinnesstamning_Värde |
| MADRS-S score 6 mos after rTMS question 2 | [number] | uppfMADRSSOroskanslor_Värde |
| MADRS-S score 6 mos after rTMS question 3 | [number] | uppfMADRSSSomn_Värde |
| MADRS-S score 6 mos after rTMS question 4 | [number] | uppfMADRSSMatlust_Värde |
| MADRS-S score 6 mos after rTMS question 5 | [number] | uppfMADRSSKoncformaga_Värde |
| MADRS-S score 6 mos after rTMS question 6 | [number] | uppfMADRSSInitiativformaga_Värde |
| MADRS-S score 6 mos after rTMS question 7 | [number] | uppfMADRSSKanslomassigtEng_Värde |
| MADRS-S score 6 mos after rTMS question 8 | [number] | uppfMADRSSPessimism_Värde |
| MADRS-S score 6 mos after rTMS question 9 | [number] | uppfMADRSSLivslust_Värde |
| MADRS-S score 6 mos after rTMS total | [number] | uppfMADRSSTotalpoang |
| CPRS-MS score 6 mos after rTMS | [number] | uppfMinnesstorning_Värde |
| EQ-5D score 6 mos after rTMS question 1 | [number] | uppfEQ5DRorlighet_Värde |
| EQ-5D score 6 mos after rTMS question 2 | [number] | uppfEQ5DHygien_Värde |
| EQ-5D score 6 mos after rTMS question 3 | [number] | uppfEQ5DHuvudsakAktiviteter_Värde |
| EQ-5D score 6 mos after rTMS question 4 | [number] | uppfEQ5DSmartorBesvar_Värde |
| EQ-5D score 6 mos after rTMS question 5 | [number] | uppfEQ5DOroNedstamdhet_Värde |
| EQ-VAS score 6 mos after rTMS | [number] | uppfEQ5DHalsotillstand |
| Persistent side effect | [y/n/m] | kvarstaendeBiverkan Värde |
| Persistent side effect | [free text] | kvarstaendBiverkanFritext |
| Hospital | [multiple choice] | sjukhus |
| Hospital (continued) | [multiple choice] | sjukhus |
| Regional council | [multiple choice] | län |
